# Task Conflict and Task Control: A Mini-Review

**DOI:** 10.3389/fpsyg.2019.01598

**Published:** 2019-07-17

**Authors:** Ran Littman, Eldad Keha, Eyal Kalanthroff

**Affiliations:** ^1^ The Clinical Neuropsychology Laboratory, Department of Psychology, The Hebrew University of Jerusalem, Jerusalem, Israel; ^2^ Department of Psychology, Achva Academic College, Arugot, Israel

**Keywords:** Stroop task, cognitive control, executive functions, task conflict, task control, stimulus-driven behavior

## Abstract

Stimulus-driven behaviors are triggered by the specific stimuli with which they are associated. For example, words elicit automatic reading behavior. When stimulus-driven behaviors are incongruent with one’s current goals, task conflict can emerge, requiring the activation of a task control mechanism. The Stroop task induces task conflict by asking participants to focus on color naming and ignore the automatic, stimulus-driven, irrelevant word reading task. Thus, task conflict manifests in Stroop incongruent as well as in congruent trials. Previous studies demonstrated that when task control fails, reaction times in congruent trials slow down, leading to a reversed facilitation effect. In the present mini-review, we review the literature on the manifestation of task conflict and the recruitment of task control in the Stroop task and present the physiological and behavioral signatures of task control and task conflict. We then suggest that the notion of task conflict is strongly related to the concept of stimulus-driven behaviors and present examples for the manifestation of stimulus-driven task conflict in the Stroop task and additional tasks, including object-interference and affordances tasks. The reviewed literature supports the illustration of task conflict as a specific type of conflict, which is different from other conflict types and may manifest in different tasks and under diverse modalities of response.

The concept of cognitive control refers to a set of abilities which allow for the effortful application and maintenance of goal-directed behaviors (Banich, 2009; Diamond, 2013). For several decades, the Stroop task has been serving as a principal tool for investigating cognitive control in the lab (MacLeod, 1991). In the present mini-review, we focus on a unique feature of cognitive control, *task control*, and its recruitment for the resolution of a specific type of conflict – *task conflict*. We first review the literature of Stroop task conflict, illustrate task conflict’s physiological and behavioral signature and then move to describe task conflict in the context of stimulus-driven behaviors, refer to its manifestation in other tasks and under diverse modalities of response, and suggest that impaired task control may be related to certain pathological behaviors.

## Task Conflict in the Stroop Task

In various situations, individuals must decide between two alternative task demands. Such circumstances often result in the emergence of task conflict. Task conflict has been studied mainly by using the Stroop task (Stroop, 1935) in which participants are instructed to name the ink-color of congruent (e.g., RED written in red), incongruent (e.g., RED written in blue), and non-word neutral (e.g., XXXX written in red) stimuli while ignoring the word’s meaning (MacLeod, 1991). The typical Stroop reaction time (RT) data show a robust Stroop interference effect (incongruent RT > neutral RT) and a smaller and less robust Stroop facilitation effect (congruent RT < neutral RT). Goldfarb and Henik (2007) suggested that the Stroop task consists of two separate conflicts – an *information conflict* between the incongruent word and ink color, which manifests in incongruent trials because of the incongruency between task-relevant and task-irrelevant *information* (e.g., blue and red); and a *task conflict* between the relevant color-naming *task* and the irrelevant, stimulus-driven word-reading task, which manifests in incongruent as well as in congruent trials because words trigger an automatic tendency to read (also see Rogers and Monsell, 1995; MacLeod and MacDonald, 2000; Levin and Tzelgov, 2016b; Kalanthroff et al., 2018a). Thus, while Stroop incongruent trials consist of both information conflict and task conflict, Stroop congruent trials consist of task conflict and not information conflict. Accordingly, the RT difference between non-word neutrals (which serve as a conflict-free baseline of general performance) and congruent conditions commonly serves as a measure of task conflict (Goldfarb and Henik, 2007; Kalanthroff et al., 2018a). Dissociation between the two conflicts was demonstrated by their diverse patterns of brain activation (Aarts et al., 2009; Desmet et al., 2011; Elchlepp et al., 2013) and their reflection in different components of an ex-Gaussian distribution (Steinhauser and Hübner, 2009; also see Aarts et al., 2009; Moutsopoulou and Waszak, 2012; Shahar and Meiran, 2015). These findings support the existence of task conflict as a specific type of conflict that is dissociated from other conflict types.

## Physiological Signature of Task Conflict and Task Control

The resolution of task conflict is managed by the activation of a task control mechanism (Entel et al., 2015; Kalanthroff et al., 2018a; Schuch et al., 2019). Neuroimaging studies have shown that the anterior cingulate cortex (ACC) – a brain area that is involved in conflict monitoring (Carter et al., 1998, 1999; Botvinick et al., 1999, 2004; Bush et al., 2000; Braver et al., 2001; Kerns et al., 2004) is more active, not only when contrasting incongruent Stroop trials to non-word neutrals but also when contrasting congruent trials to non-word neutrals (Bench et al., 1993; Carter et al., 1995; Milham et al., 2002; Aarts et al., 2009).

Recent neuroimaging studies have provided evidence for the locus of task control in the brain. These studies have manipulated task conflict by using a word-arrow version of the Stroop task (Aarts et al., 2009) or by manipulating the proportion of congruent, incongruent, and neutral trials within Stroop blocks (Grandjean et al., 2012, 2013), a manipulation that reduces or enhances task control (see below). The data from these studies (Aarts et al., 2009; Grandjean et al., 2012, 2013) support the idea that task conflict results in activation of the ACC, the medial superior frontal gyrus (MFC), and ventral areas of the lateral prefrontal cortex (L-PFC). Subsequently, the resolution of task conflict is reflected by an involvement of the dorsal part of the L-PFC (DL-PFC), which marks the top-down monitoring processes of favoring the relevant task and the implementation of task demands (MacDonald et al., 2000; Egner and Hirsch, 2005; Carter and Van Veen, 2007; Brosnan and Wiegand, 2017). Additional findings marked the differences in brain activation in the face of task conflict and information conflict. While both conflicts activated the ACC and the MFC, information conflict was associated with activity in ventral L-PFC, whereas task conflict activated both ventral and dorsal regions (Aarts et al., 2009).

Other studies have employed Stroop tasks while scrutinizing changes in pupil dilation, which has been used as a measure of effort extraction and the employment of cognitive control (Kahneman and Beatty, 1966; for reviews see Beatty and Lucero-Wagoner, 2000; Laeng et al., 2012; Sirois and Brisson, 2014; van der Wel and van Steenbergen, 2018). These studies provided evidence for interference and facilitation effects, measured by pupil dilation (Brown et al., 1999; Siegle et al., 2004, 2008; Laeng et al., 2011; Hasshim and Parris, 2015). Recently, Hershman and Henik (in press) reported a dissociation between task conflict and information conflict by measures of pupil dilation. Specifically, participants’ pupils became dilated when observing both congruent and incongruent trials in comparison to non-word neutrals at about 500 ms after the stimulus onset. A second dilation became evident for incongruent trials only at about 900 ms after the stimulus onset. These data show that the emergence of task conflict (and the recruitment of task control) precedes the emergence of information conflict and support previous suggestions after which the presentation of two task sets lead to the emergence of task conflict even before information regarding stimulus’ identity of dimensions begins to compute (MacLeod and MacDonald, 2000; Monsell et al., 2001; Goldfarb and Henik, 2007; Steinhauser and Hübner, 2009; Braverman et al., 2014).

## Behavioral Signature of Task Conflict and Task Control

**Figure 1:**
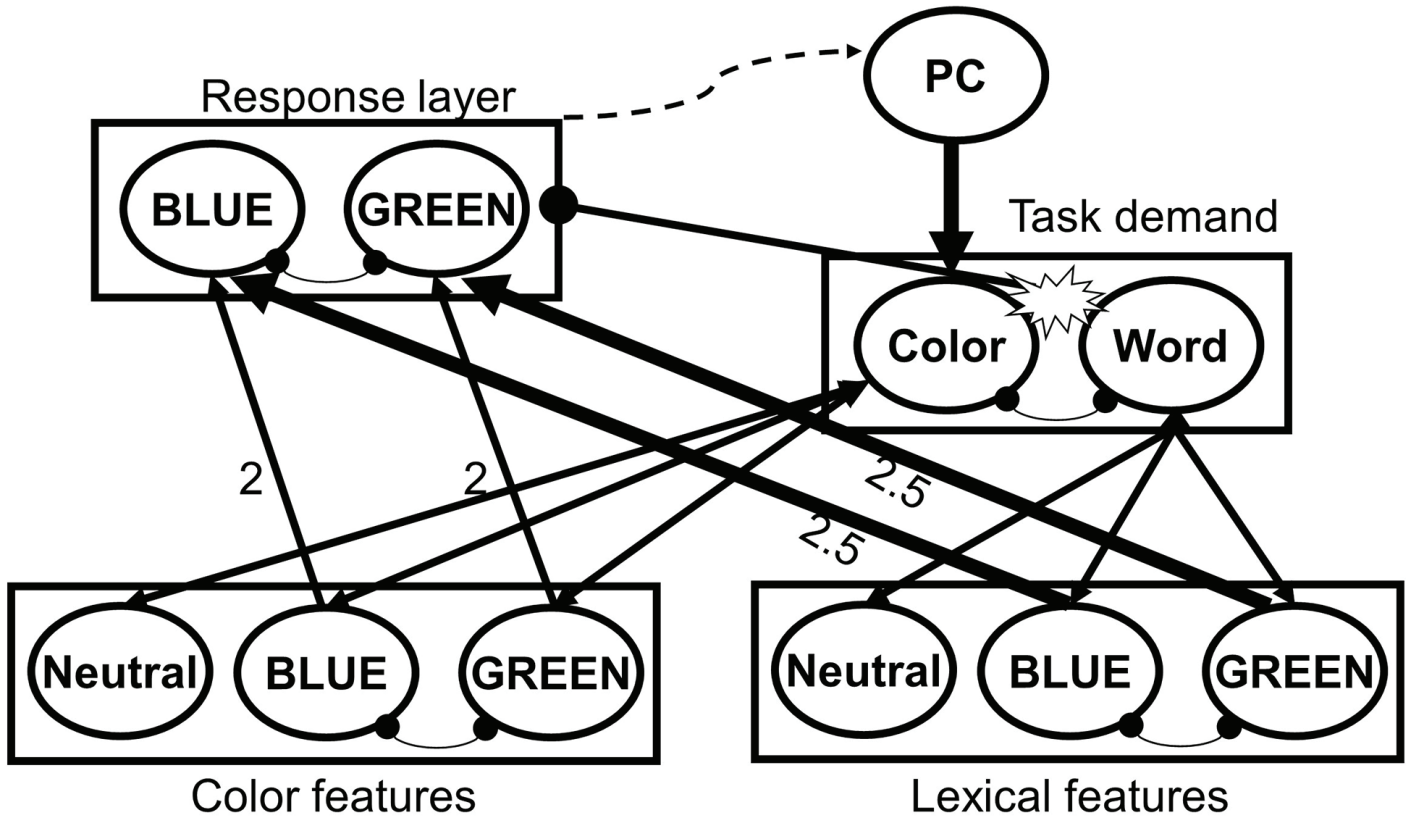
Architecture of the proactive control/task conflict (PC-TC) model of the Stroop task. From Kalanthroff et al., 2018a, p. 2. Copyright 2018 by American Psychological Association. Reprinted with permission from American Psychological Association. In this model, task control is considered a proactive, effortful process that deploys control in advance of the stimulus for the resolution of conflict (De Pisapia and Braver, 2006; Braver et al., 2007; Barch and Ceaser, 2012; Braver, 2012). Pointy-headed arrows represent excitatory connections, whereas the round-headed arrows represent inhibitory connections. A stimulus activates its color and lexical representations in the input (features) layers. The activations from the input layers propagate to the response layer and to the task demand layer, which feeds back to the input layers. Congruent and incongruent color words, but not (non-word) neutral stimuli, activate both task demand units, which lead to task conflict. This task conflict inhibits the response layer, thereby slowing down responses to color words and resulting in Stroop reverse facilitation effect. When proactive control is high, attention is sufficiently biased in a top-down manner to the color-naming task demand unit, thus preventing (or rapidly resolving) task conflict and resulting in Stroop facilitation effect. However, manipulations that reduce proactive control lead to a stronger capture of attention by the irrelevant task dimension (word meaning), resulting in a reverse facilitation effect. This process takes place in both congruent and incongruent trials. In incongruent trials, an additional information conflict takes place when both input layers provide contradictory information (e.g., blue in the color features and green in the lexical features), leading to the activation of the two (mutually inhibitory) response units in the response layer, which causes the slowing down of reaction time and result in a (robust) Stroop interference effect.

The physiological evidence for the emergence of task conflict in Stroop congruent trials appears to stand in contradiction with behavioral findings, which indicate that responses to congruent trials are often faster than to neutral trials. It has been suggested (Goldfarb and Henik, 2007; Kalanthroff et al., 2018a) that in healthy adults, task control is highly efficient and leads to a rapid resolution of task conflict. Hence, task conflict is not behaviorally observable under standard conditions but can be seen under specific conditions, yielding in Stroop reverse facilitation (RF; faster responses to neutral stimuli than to congruent stimuli), which serves as the behavioral signature of task conflict (Kalanthroff et al., 2018a). For example, to illustrate Stroop RF, several studies have manipulated the proportion of congruent, incongruent, and neutral trials, creating blocks that consist of a majority or a minority of non-word neutrals, a manipulation that reduces or enhances task control, respectively, as participants mostly encounter non-conflictual or conflictual trials (Tzelgov et al., 1992; Goldfarb and Henik, 2007; Kalanthroff et al., 2013c; Entel et al., 2015; Shichel and Tzelgov, 2018). Other studies presented a cue that indicated whether the following trial will be conflictual or not (Goldfarb and Henik, 2007), have manipulated the length of the response-stimulus interval (RSI; Parris, 2014), or combined the Stroop task with additional measures of working memory (Kalanthroff et al., 2015), inhibitory control (Kalanthroff and Henik, 2013; Kalanthroff et al., 2013b), and task switching (Kalanthroff and Henik, 2014). The accumulating evidence from these studies shows that, when task control is overloaded, or, alternatively, when task control is reduced and “put to sleep,” Stroop RF, signifying the behavioral marker of task conflict, becomes evident (however see Augustinova et al., 2018, for different results when using an RSI procedure). Recently, Kalanthroff et al. (2018a) have presented a computational model of the Stroop task, the proactive control/task conflict (PC-TC) model, which illustrates the resolution of task conflict and its modulation by task control (Figure 1). This model extends a previous model of the Stroop task (Botvinick et al., 2001) by accounting for the effects of task conflict and predicting RF. Behavioral evidence of task conflict was also demonstrated in task-switching paradigms (Braverman and Meiran, 2010; Schneider, 2015; Bugg and Braver, 2016), where a cue indicates which of two pre-determined tasks the participant needs to execute during a given trial. Unlike the Stroop task, in task-switching paradigms both tasks are relevant to some extent and the controlled process of favoring the relevant task cannot be prepared in advance.

The evidence discussed above illustrates task control as a specific type of cognitive control mechanism, which is recruited to resolve a specific type of conflict, task conflict. In the following section, we suggest that the emergence of task conflict and the recruitment of task control are strongly related to the concept of stimulus-driven behaviors.

## Task Conflict in the Context of Stimulus-Driven Behaviors

Stimulus-driven behaviors are triggered by the specific stimuli with which they are associated (Monsell, 2003; Waszak et al., 2003; Koch and Allport, 2006; Reuss et al., 2011; Ganor-Moscovitz et al., 2018; Hochman et al., 2018). This concept has been widely investigated outside the scope of the task-control framework, and it echoes the findings of instrumental conditioning in animal studies: After an association between a stimulus and an action was established, animals were shown to keep responding to the stimulus even when it no longer predicted a reward and demonstrated spontaneous recovery of the stimulus-response (S-R) association even after undergoing extinction (Graham and Gagné, 1940; Guttman, 1953; Skinner, 1953; Rescorla, 1993; Bouton, 2004). In humans, several studies have demonstrated the automatic triggering of response activation processes when facing stimuli which were associated with certain responses, even when these responses were not eventually executed (Osman et al., 1992; De Jong et al., 1994; Eimer, 1995; Valle-Inclán, 1996; Gibbons and Stahl, 2008; also see Rothermund et al., 2005).

The concept of S-R binding is relevant to the processes taking place in the Stroop task (Mordkoff, 1996; Schmidt et al., 2007; Schmidt and Besner, 2008), where words elicit automatic reading behavior, even without an explicit intention to read (MacLeod and MacDonald, 2000; Monsell et al., 2001; Perlman and Tzelgov, 2006; Augustinova and Ferrand, 2014). Consequently, when the stimulus-driven reading behavior is incongruent with one’s current goals, task conflict between stimulus-driven and goal-directed behaviors emerges, requiring the activation of a task control mechanism for the resolution of conflict (Kalanthroff et al., 2018a). Hence, in both congruent and incongruent Stroop conditions, stimulus-driven task-irrelevant word reading is incongruent with the relevant task of color naming, leading to the emergence of task conflict. Importantly, interference due to task conflict can manifest as long as the stimulus can be read, regardless of whether it is color related or not (Levin and Tzelgov, 2014, 2016a). Hence, non-color word neutrals (e.g., CHAIR in red) and pseudo words (e.g., HIX) also trigger the stimulus-driven reading behavior and result in the emergence of task conflict (Monsell et al., 2001; Goldfarb and Henik, 2007; Kinoshita et al., 2017; Kalanthroff et al., 2018a). The following examples illustrate the manifestation of stimulus-driven task conflict in different tasks and under diverse modalities of response in addition to the Stroop task.

Following the notion that form-based object-naming and classification is habitual and automatic in children (Kagan and Lemkin, 1961; Siegel and Vance, 1970; Bloom, 2002; Diesendruck and Bloom, 2003), Prevor and Diamond (2005) have used a color-object Stroop task, asking young children to name the colors of abstract shapes and familiar objects, which were presented in their congruent (e.g., a yellow banana), incongruent (e.g., a blue banana), or neutral (e.g., a purple scissors) colors. Because of their stimulus-driven tendency to name the objects, children were slower and less accurate in naming the color of namable objects in comparison to abstract forms, even when the objects appeared in their congruent colors. In a series of studies, La Heij and colleagues have replicated and elaborated these findings (La Heij et al., 2010; La Heij and Boelens, 2011, 2013; also see Starreveld and La Heij, 2017). Specifically, the authors demonstrated that the “object-interference effect” manifests due to the competition between the task set of color naming and the children’s stimulus-driven prepotent tendency to name the object and not by other types of conflicts, such as lexical-based response conflict (La Heij et al., 2010; La Heij and Boelens, 2011). These findings implicate a stimulus-driven task conflict, which resembles the task conflict taking place in the Stroop task, manifesting in children who are unable to read.

Recently, we have investigated the emergence of task conflict in an affordance task. According to Gibson’s (1979) theory of affordances, a common manipulatable object may trigger a response that has acquired a strong association with it (Rogers and Monsell, 1995; Allport and Wylie, 2000). Thus, simply viewing a manipulatable object triggers automatic and specific motor plans for interacting with it, even in the absence of an explicit intention for interaction (Vainio et al., 2008; Makris et al., 2013), as is evident by the automatic activation of the pre-motor cortex (Martin et al., 1996; Creem-Regehr and Lee, 2005; Beauchamp and Martin, 2007; Proverbio et al., 2011, 2013; Righi et al., 2014). In affordance tasks, participants are asked to classify objects (e.g., natural vs. manufactured) by responding with their left or right hand. The objects are presented as to trigger an automatic grabbing response in one hand (e.g., a cup with the handle turning rightwards), and the participants must suppress their automatic tendency of grabbing the object by its extended handle. Participants typically respond faster and more accurately when the relevant response (classifying the object) and the automatic, task-irrelevant response (grabbing the object) result in the activation of the same hand rather than different hands (Tucker and Ellis, 1998, 2004; Ellis and Tucker, 2000; Phillips and Ward, 2002; Tipper et al., 2006; Vainio et al., 2007; Pellicano et al., 2010). Recent data from our lab show that the resolution of task conflict in the Stroop task strongly predicted the resolution of conflict in the affordance task level (grab the object vs. classify the object), but not in the affordance response level (responding with the right hand vs. left hand; Littman & Kalanthroff, manuscript in preparation). These findings link the emergence of stimulus-driven task conflict in both tasks, indicating the operation of a shared task control mechanism. As the Stroop task is based on linguistic skills and the affordance task calls for the activation of visuomotor abilities, these findings also illustrate the emergence of task conflict (and the recruitment of task control) in different tasks and under diverse modalities of response.

Recently, the conceptualization of task conflict as the result of stimulus-driven behaviors has proven to be an efficient framework for the understanding of several pathologies (Kalanthroff et al., 2018a). For example, it has been proposed that compulsivity in obsessive-compulsive disorder (OCD) may be strongly connected to excessive stimulus-response habit formation, rendering patients’ capability of following elaborated environmental models in a manner that supports goal-directed behavior (Robbins et al., 2012; Kalanthroff et al., 2013a, 2018b; Gillan et al., 2014, 2015). In line with the task conflict framework, failure to suppress irrelevant stimulus-driven behaviors as a result of reduced task control functioning was suggested to be a pathological trait that also constitutes a core characteristic of the inability to suppress compulsive behaviors (Kalanthroff et al., 2017, 2018b). Following this line of study, interventions for the amelioration of task control abilities may prove useful for the enhancement of OCD patients’ capability to suppress their urges to engage in compulsive behaviors.

## Conclusion

In the present work, we have reviewed the literature of task conflict, which manifests when several, contradictory task sets are activated simultaneously. The accumulating evidence aid portraying task conflict as a unique feature of cognitive control, which is distinct from other conflict types and results in specific neuronal and behavioral signatures. Task conflict has been shown to manifest under the Stroop task and additional tasks including task switching, object interference, and affordance tasks, and to be strongly related to the concept of stimulus-driven behaviors.

One final note should be mentioned. Despite the ample evidence for the manifestation of different conflict types in the Stroop and Stroop-like tasks (Kornblum, 1992, 1994; Kornblum and Lee, 1995), some researchers who are interested in Stroop interference seem to neglect that it goes beyond response competition or ignore the (non-word) neutral condition and use the RT difference between congruent and incongruent conditions as a sole measure. These practices may lead to overlooking some important aspects of cognitive control and result in misinterpretations of certain results (Augustinova et al., 2018; Hershman and Henik, in press). To avoid such errors, the contribution of task conflict to the general Stroop conflict should be regularly considered.

## Author Contributions

All authors listed have made a substantial, direct and intellectual contribution to the work, and approved it for publication.

### Conflict of Interest Statement

The authors declare that the research was conducted in the absence of any commercial or financial relationships that could be construed as a potential conflict of interest.
